# Surgical resection in medulloblastoma: a crossroads in the molecular era

**DOI:** 10.1007/s00381-026-07355-0

**Published:** 2026-07-08

**Authors:** Joseph H. McAbee, David G. Laird, Amar Gajjar, David S. Hersh, Nir Shimony, Kristian Aquilina, Paul Klimo

**Affiliations:** 1 Surgical Neurology Branch, NINDS, National Institutes of Health, Bethesda, MD USA; 2 College of Medicine, University of Tennessee Health Science Center, Memphis, TN USA; 3 St. Jude Children’s Research Hospital, Memphis, TN USA; 4 Division of Neurosurgery, Connecticut Children’s, Hartford, CT USA; 5 Department of Neurosurgery, UConn School of Medicine, Farmington, CT USA; 6 Department of Neurosurgery, University of Tennessee Health Science Center, Memphis, TN USA; 7 Neuroscience Institute, Le Bonheur Children’s Hospital, Memphis, TN USA; 8 Semmes Murphey Clinic, Memphis, TN 38120 USA; 9 Department of Neurosurgery, Great Ormond Street Hospital for Children, London, UK

**Keywords:** Extent of resection, Medulloblastoma, Near total resection

## Abstract

Surgical resection for medulloblastoma has been a mainstay of treatment for decades due to the need for definitive diagnosis and known benefits on treatment efficacy and survival. While many pediatric neurosurgeons and neurooncologists agree that maximal safe surgical resection should be the goal of medulloblastoma surgery, actual practical application and benefits of maximal safe resection seem to vary. Early work on extent of resection provided a relatively arbitrary 1.5 cm^2^ threshold to distinguish subtotal resection (STR) from near total resection (NTR) and demonstrated a survival benefit if the residual was ≤ 1.5 cm^2^ (NTR or gross total resection (GTR)). However, recent evidence questions the validity of this threshold, with European researchers even suggesting that STR alone should not be considered a high-risk feature during adjuvant therapy planning. The question regarding extent of resection, therefore, is undeniably controversial and has become even more complex in recent years due to the seminal work done to identify medulloblastoma subgroups based on molecular characteristics. Adjuvant treatment approaches are being tailored to these molecular subgroups, and the impact of extent of resection on survival is being reconsidered within the context of subgroups. Certainly, the goal when treating children with medulloblastoma, or any form of brain cancer, should be to cure the disease while minimizing treatment-related toxicity, including surgical injury. In this article, we discuss the role of surgical resection for treating medulloblastoma and whether it is possible to maximize both surgical resection, to potentially improve survival and adjuvant treatment efficacy, while minimizing neurological morbidity.

## History of surgical resection for medulloblastoma

Medulloblastoma is the most common malignant primary brain tumor in children [18]. It is estimated that approximately one-third of children with medulloblastoma eventually succumb to their disease, while those who survive often experience significant morbidity related to disease progression and treatment effects [23]. The Children’s Cancer Group 921 study (CCG-921) was conducted in the 1980 s and 1990 s to examine the correlation between extent of medulloblastoma resection and prognosis in 188 patients. In addition to overall survival (OS) benefit for older children (older than 3 years) and patients with non-metastatic disease, CCG-921 demonstrated a progression-free survival (PFS) advantage for lower residual tumor burden [1, 31]. The study established 1.5 cm^2^ as the threshold for residual tumor that distinguishes near-total resection (NTR) from sub-total resection (STR), based predominately on CT imaging. For the first time, a quantifiable imaging-based measurement was put forth as a “gold standard” for the evaluation of surgical resection, rather than relying on the often inaccurate intraoperative observations of the neurosurgeon. However, this threshold was not established by an a priori hypothesis but instead was reached through an iterative statistical process that resulted in statistically significant separation of the survival curves [1, 31]. The authors noted that residual tumor ≤ 1.5 cm^2^ on postoperative scans could significantly improve PFS, likely because a smaller tumor burden is more amenable to effective control by adjuvant therapy. Other subsequent studies—such as the International Society of Pediatric Oncology (*Société internationale d'oncologie pédiatrique* or SIOP) Primitive Neuroectodermal Tumors study (PNET 4, 2012) which compared hyperfractionated radiotherapy with standard radiotherapy and the Children’s Oncology Group (COG) Phase III trial (ACNS033, 2021) which evaluated reduced dose and volume radiotherapy with chemotherapy—corroborated that residual disease in excess of 1.5 cm^2^ was associated with a worse outcome [13, 15].

Since the above-mentioned trials were conducted, this controversial threshold has remained the standard for clinical decision-making. However, the limitations of these trials coupled with potentially greater morbidity from more aggressive resections have led some groups to question whether the 1.5 cm^2^ “rule” retains its value in the new era of molecular subgroups and targeted therapies [28].

## Medulloblastoma molecular subgroups

Since the conclusion of the CCG-921 trial in 1992, there have been many major advances in the field of neurosurgical oncology, particularly for medulloblastoma. Rapid advances in magnetic resonance imaging (MRI) technology coupled with the subspecialization of pediatric neuroradiologists have dramatically improved the imaging evaluation of medulloblastomas. Surgical techniques, instruments, and pediatric neuroanesthesia continue to be refined. Intraoperative technology, such as ultrasound and intraoperative MRI, has provided surgeons with the means to assess extent of resection in real time. Importantly, the medulloblastoma community experienced a seismic shift in 2012 with the landmark publication by Taylor et al. that delineated medulloblastomas into subgroups based on transcriptional profiling, genetic mutation patterns, and clinical characteristics rather than relying solely on histologic features [26]. The initial medulloblastoma subgroups included wingless/INT1-activated (WNT), sonic hedgehog-activated (SHH), group 3, and group 4. The most recent iteration of the World Health Organization (WHO) Classification of Tumors of the Central Nervous System (CNS) in 2021 utilized distinct clinical and biological differences, and incorporated wide-scale methylation profiling results, to develop the current state of medulloblastoma classification: medulloblastoma, WNT-activated; medulloblastoma, SHH-activated *TP53* wildtype; medulloblastoma, SHH-activated *TP53* mutant (further divided into 4 SHH subgroups based primarily on methylation); and medulloblastoma, non-WNT/non-SHH (formerly groups 3 and 4, now further divided into 8 subgroups) [14, 24].

While further changes are likely to occur in future classification systems, medulloblastoma subgroups have now become integrated into prognostication, as well as in the development and application of targeted, risk-stratified therapeutic strategies [3, 5, 16, 25]. Despite the explosion of molecular information, the specific impact of extent of surgical resection on each individual subgroup is largely unknown. An unintended consequence of the molecular classification of medulloblastoma—already a relatively rare tumor—into multiple subgroups and subtypes is that the number of patients within each individual classification becomes smaller and smaller, which makes determining the effect of any intervention, including surgery, harder to evaluate. Furthermore, potential confounders, such as age and the presence of metastatic disease, may also contribute disproportionately from group to group. As a result, specific clinicopathological characteristics and the natural history of individual subgroups may affect the risk-benefit analysis for surgery. For example, given their excellent prognosis, children with medulloblastomas in the WNT subgroup may not require the surgical resection to be as aggressive as for medulloblastomas in other subgroups. However, in patients with a newly diagnosed posterior fossa tumor, preoperative imaging may not even accurately predict the general histopathological diagnosis of medulloblastoma, let alone the specific molecular subgroup. Therefore, information regarding the medulloblastoma subgroup is currently unable to influence surgical decision-making for a primary resection. This issue may be settled in the future with ongoing advances in nanopore sequencing, which facilitates a rapid intraoperative diagnosis [4, 8, 19]. Ultimately, such technology coupled with effective non-operative therapy for specific molecular subgroups may allow a paradigm shift to biopsy or STR (coupled with hydrocephalus management). Until then, however, surgical planning must proceed in the absence of molecular data.

## Ongoing controversy—different strategies based on institution and region

**Fig. 1:**
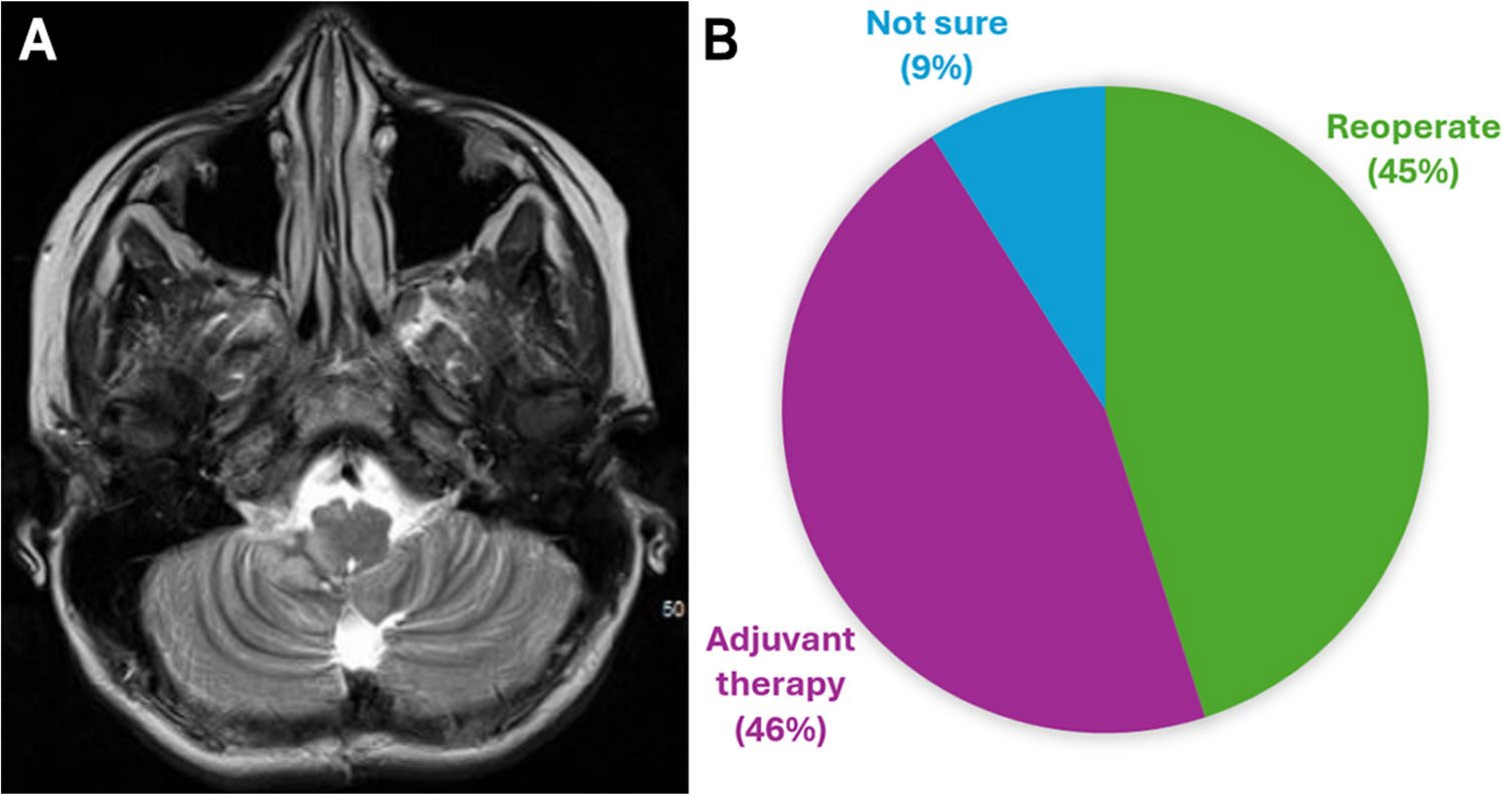
**A** A small residual medulloblastoma (2.0 × 1.4 cm) in right cerebellomedullary angle. **B** Summary of responses to a poll administered at the 2020 AANS/CNS Pediatric Section Meeting that asked respondents about their preferred treatment strategy for such a lesion

While our molecular knowledge of medulloblastoma has advanced tremendously, the question of the role of resection has become ever more complicated and vexing [21]. As we move closer to having longer survival data available for patients treated in the molecular subgroup era with different targeted strategies, it appears we are currently at a crossroads concerning the extent of surgical resection in pediatric medulloblastoma patients—do surgeons strive for maximal resection, ideally with a residual of less than 1.5cm^2^, or do they debulk or even just biopsy and proceed with adjuvant therapy? This dilemma is highlighted not just by alternative surgical opinions from the literature across different geographic locations, but also by a poll conducted at the 2020 joint American Association of Neurological Surgeons/Congress of Neurological Surgeons (AANS/CNS) Pediatric Section Meeting. The audience was asked what their preferred intervention would be for a pediatric patient found to have a 2.0 cm residual lesion in the right cerebellomedullary angle (Fig. 1A). An almost equivalent number of attendees responded that they would reoperate versus leaving the residual and starting adjuvant therapy (Fig. 1B).

A retrospective study by Thompson et al. of 787 patients with medulloblastoma treated at 35 international institutions found a PFS benefit when comparing gross total resection (GTR) with STR but found no differences in PFS or OS when comparing GTR with NTR [28]. Furthermore, when analyzing based on subgroups, only the group 4 subgroup had a PFS survival advantage after GTR. The authors argued that extent of resection for certain subgroups could be limited to avoid morbidity and suggested leaving residual disease behind that is adherent to critical structures. It should be noted that extent of resection was based on non-centralized, institution-specific radiologist reads of postoperative T1 MRIs. In addition to the retrospective study, Thompson et al. undertook a systematic review of studies related to extent of resection in medulloblastoma and found similar studies supporting (*n* = 16) or refuting (*n* = 20) an association between extent of resection and survival [27]. Fourteen studies had mixed results, while only three articles included molecular subgroup analysis. There were significant limitations in the 50 studies that were included in their analysis. Articles were published across a 40-year time span, from 1977 to 2017. All articles were retrospective either in analysis alone or in both data collection and analysis. There was no consensus on how extent of resection was assessed or categorized, including some that were based solely on surgeons’ reports. Given the results of these two important studies by Thompson and colleagues, what is clear is that there is no definitive answer about the prognostic significance of extent of resection.

In the USA and Canada, children with medulloblastoma are often considered high-risk if they present with metastases at diagnosis, are younger than 3 years of age, and/or have residual disease ≥ 1.5 cm^2^. We surmised from the findings of the CCG-921 study, as well as from collective experience with other brain tumors in which increased extent of resection generally yields improved survival, that achieving a GTR/NTR when safely feasible is still the surgical goal in medulloblastoma at most high-volume pediatric institutions in North America. Depending on the institution, some high-risk medulloblastoma patients with unequivocal or suspected residual tumor (especially if the residual tumor is ≥ 1.5 cm^2^) may undergo a second-look surgery or be treated according to high-risk protocols with intensified chemoradiotherapy regimens [20, 28].

In recent years, researchers in the UK and Europe have questioned the validity of STR (i.e., > 1.5 cm^2^ of residual tumor) as a high-risk disease feature. In fact, Simon Bailey and coauthors state that, “extent of surgical resection is no longer considered a prognostic variable in medulloblastoma,” citing several primarily European studies [2]. For example, in the European SIOP High-Risk Medulloblastoma (SIOP-HR-MB) clinical trial for non-infant, high-risk medulloblastoma, STR was not considered a high-risk feature in the absence of other high-risk features: metastatic disease, large cell/anaplastic histology (LCA), *MYC* amplification, *TP53* amplification, or *MYCN* amplification in SHH tumors [2, 11]. In their retrospective cohort of 1100 patients (416 from UK Children’s Cancer Center, and the remainder from previously published cohorts), Keeling et al. found that STR (*n* = 226, 20%) predicted lower overall survival compared with GTR (*n* = 884, 80%) [11]. However, in their multivariable analysis, STR was not found to be an independent prognostic factor across all subgroups when examined alongside other high-risk features. This led to the assertion that patients with STR should be considered as standard risk (and not high risk) for adjuvant treatment planning and clinical trial inclusion purposes [11, 28]. While this UK analysis was said to be independent of adjuvant treatment effects, it is highly likely, as noted by the authors, that the majority of STR patients underwent more intensive treatment regimens (since STR was considered a high-risk feature at the time), thus potentially complicating the findings. Furthermore, STRs in the UK cohort were more likely to be encountered in younger patients and non-WNT patients. This higher preponderance of STR in patients who were already at higher risk of poor outcomes could serve as a confounder. Lastly, a previous study by the same group demonstrated improved overall survival among patients who underwent re-resection at the time of relapse [10]. The demonstration of a survival advantage for re-resection at relapse might be extrapolated to the primary resection as well, suggesting a benefit of more aggressive resections rather than STR. We await the results from the SIOP-PNET5-MB trial, a European trial (concluded in 2022) that used clinical, histological, and molecular parameters for children and adolescents with standard-risk medulloblastoma [17].

Another surgical opinion stems from Egypt. Enayet et al. retrospectively studied 405 patients with medulloblastoma treated in Egypt [7]. The 5-year overall survival was reported to be 79.5% for STR versus 87.6–96.3% for GTR/NTR, while the 5-year PFS was 70.1% for STR versus 83.9–86.1% for GTR/NTR. Ultimately, the authors found no significant OS (*p* = 0.557) or PFS (*p* = 0.146) benefit between GTR/NTR or STR. The study also found no association between GTR and increased risk of complications, like posterior fossa syndrome; the authors therefore concluded that GTR should still be pursued whenever possible. Of note, these patients were not stratified by molecular subgroup, but age and disease dissemination were considered. The group at Dana Children’s Hospital in Tel Aviv has recently offered some thought-provoking results in patients presenting with metastatic disease [6]. Twelve children with a mean age of 6.5 years (range, 1.1–16.1) underwent biopsy only (with or without shunt), followed by adjuvant therapy. Three children under the age of 3 were treated with chemotherapy only; the remaining 9 patients had craniospinal radiation with chemotherapy. At last follow-up (median, 3.2 years), 9 (75%) were alive with the following molecular subgroups: SHH (*n* = 4), group 3 (*n* = 2), group 4 (*n* = 2), and unknown (*n* = 1). Estimated 5-year survival was 65%. Of the 3 children who died, 2 had MYC-amplified group 3 tumors and 1 was unknown. The authors concluded that this minimalistic approach should be considered in these high-risk patients.

## The St. Jude experience

At the present, the philosophy at St. Jude Children’s Research Hospital (SJCRH) has been to strive for GTRs for all medulloblastoma patients, when possible, even those with mild-to-moderate metastatic disease. Our surgical goal is to achieve no measurable disease at the primary site prior to adjuvant therapy. Since many medulloblastoma patients come to SJCRH after their initial resection, about a third or more undergo second-look surgery for residual tumor. There are several scenarios where we would likely *not* take a patient back to the operating room for residual tumor: (1) heavy/bulky multifocal metastatic disease; (2) small amount of highly favorable (e.g., WNT) or highly unfavorable (e.g., SHH TP53 mutant, group 3 MYC amplified) molecular subgroups; (3) high risk of causing a significant and potentially irreversible neurologic deficit (e.g., tumor infiltration into eloquent structures like the facial colliculus, dentate nucleus or cerebellar peduncles); and (4) if the patient’s neurologic function was severely compromised from the initial resection (e.g., severe posterior fossa syndrome). Additionally, in some cases, it may be reasonable to perform a planned staged approach whereby an immediate decompression of the brainstem and opening of the CSF pathways is performed first, followed later by a second-look surgery. Second-look surgery may sometimes occur after neoadjuvant chemotherapy to shrink the residual tumor. It should be emphasized that each patient is thoroughly vetted in a multidisciplinary fashion.

Postoperative changes, blood products, and hemostatic agents can make determination of residual disease quite difficult. In a study of our experience with performing second-look surgery for medulloblastoma, we found that the overwhelming majority of patients had histopathologically confirmed residual tumor identified during the second-look surgery, even when the initial postoperative scan was indeterminate [20]. The most common sites of residual tumor were in lateral regions (lateral recess and/or foramen of Luschka, 67%) and the fourth ventricular roof (superior medullary velum, 76%).

**Fig. 2:**
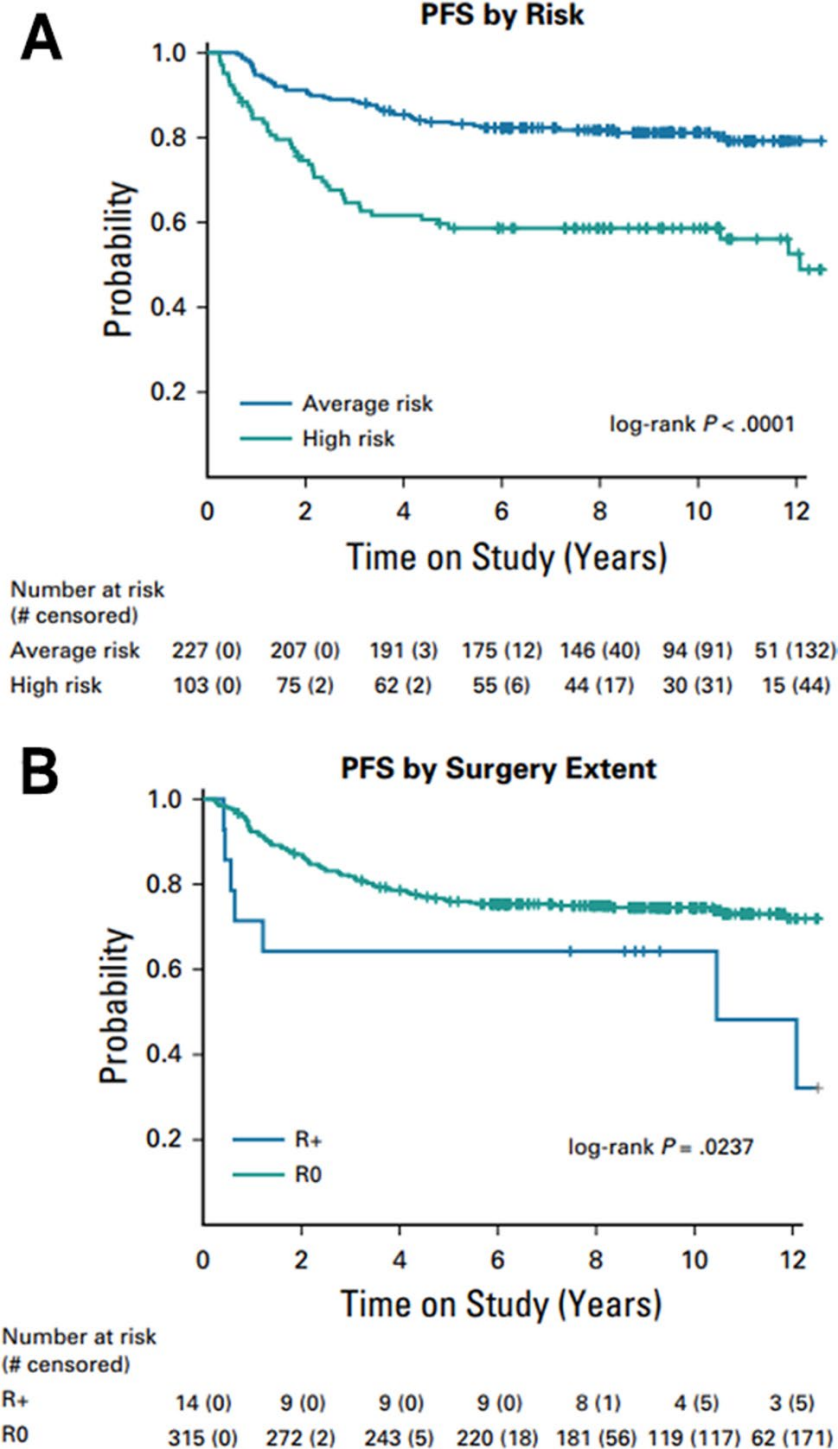
Progression-free survival based on **A** risk and **B** extent of resection (R+=STR; R0=GTR/NTR). Reproduced with permission from Gajjar A. et al. (J Clin Oncol, 2021) [9]

The St. Jude Treatment of Patients With Newly Diagnosed Medulloblastoma, Supratentorial Primitive Neuroectodermal Tumor, or Atypical Teratoid Rhabdoid Tumor (SJMB03) prospective trial, performed from 2003 to 2011 and involving 330 patients with medulloblastoma, found that extent of resection remained a significant clinical risk factor. Out of the 330 patients, 315 (95.5%) had either a GTR (*n* = 246) or NTR (*n* = 69); only 13 patients had STR and 1 patient had biopsy. In comparison, the retrospective UK study by Keeling et al. (*n* = 1110) had a GTR rate of 80% (884/1110) with the remaining patients (*n* = 226) had STR. The St. Jude study demonstrated that average-risk patients (including those with NTR) achieved 5-year PFS of 83.2%, while high-risk patients (including those with prior STR) had a 5-year PFS of 58.7% (Fig. 2A) [9]. For the overall cohort in the St. Jude study, the 5-year PFS and overall survival were 75.6% and 82.3%, respectively. In the UK study, the 5-year overall survival was 67.6%. Because extent of resection was a major driver of risk classification, the SJMB03 trial highlights that maximal safe resection may alter a patient’s overall risk due to lower disease burden and less potential therapeutic side effects from less intensive adjuvant therapy regimens, while also leading to more favorable outcomes (Fig. 2B). Similarly, the St. Jude Risk-Adapted Therapy for Young Children with Medulloblastoma (SJYC07) study, a Phase II trial for children less than 3 years old from 2007 to 2017, incorporated extent of resection, among other features, into the risk-stratification criteria [22]. The low-risk group contained patients with a GTR or NTR (defined as a residual of < 1cm^2^) whereas the intermediate group had STR. Of the 81 enrolled patients, 68 (84%) had GTR or NTR; the rest (16%) had STR. While event-free survival did not improve compared to historical controls in this trial, the low risk group did display improved survival over intermediate and high-risk groups.

## Maximizing resection and minimizing risk—surgical strategies

Optimizing surgical technique, utilizing available intraoperative technology (e.g., intraoperative MRI, intraoperative ultrasonography), and incorporating close neuromonitoring can help mitigate the risk of neurological morbidity even during more aggressive resections. A feared but unfortunately still common neurologic morbidity associated with GTR or NTR of posterior fossa tumors is cerebellar mutism syndrome (CMS)/posterior fossa syndrome (PFS). This syndrome is characterized by reduced or absent speech, emotional and behavioral lability, cerebellar dysfunction, dysphagia, and other motor signs, typically developing between 48 and 72 hours postoperatively. It is a direct result of manipulation of the proximal efferent cerebellar pathway (pECP) [29]. In some studies, CMS is estimated to affect at least 20% to 40% of children following medulloblastoma resection [30]. While there is evidence of certain non-modifiable risk factors, such as young age, a large midline tumor, and medulloblastoma pathology, there are surgical strategies and techniques that can reduce the risk of CMS.

The recent Posterior Fossa Society consensus statement on reducing the risk of CMS suggests that surgeons can improve outcomes through a number of strategies [29], including (1) using the telovelar approach; (2) using the ultrasonic aspirator and bipolar coagulation with caution, particularly at the tumor-brain interface; (3) knowing and respecting the ependymal boundaries of the 4th ventricle; (4) limiting brain retraction; and (5) avoiding critical structures, such as the floor of the 4th ventricle, the dentate nuclei, and the superior cerebellar peduncles, even when they are infiltrated by the tumor. Carefully reviewing preoperative imaging with neuroradiology is helpful in identifying areas of tumor that may be invading critical structures. While neurologic changes that occur with CMS do improve with time, the more severely the child is affected at the onset, the more likely the child will have permanent deficits once the neurologic recovery phase has plateaued. Permanent deficits may also occur independent of CMS, notably when there is violation of the ependyma through the floor of the 4th ventricle or around the cerebral aqueduct. Ultimately, while the location of medulloblastoma may put patients at risk for neurologic morbidity, we believe that with proper planning and technique, good surgical and oncologic outcomes can both be achieved.

## Putting it all together

The most reliable and currently available data are compromised by an over-reliance on the 1.5cm^2^ threshold, a number that has no biological significance and was only derived through statistical analysis. In addition, the studies themselves were not primarily designed to demonstrate the impact of extent of resection; in all cases, this was derived from analysis of secondary endpoints. As a result, while the inconsistent and at times conflicting outcomes of these individual studies are appreciated, stratification of their value and importance is difficult. While the use of intraoperative molecular pathology may eventually be helpful, its application to surgical decision-making remains questionable in the absence of clear data on the impact of extent of resection for each subgroup. The rarity of medulloblastoma, the number of subgroups and subtypes, the increasing number of adjuvant treatment protocols, and the wide variation in surgical experience and ability and reported complication rates make a randomized surgical study focused on extent of resection completely impractical.

Those surgeons who advocate for maximal resection (including potential second-look surgery) do so because they feel that it positively impacts overall survival and PFS, but that it must be done with a low risk of incurring major surgically induced, long-term neurologic deficits. These surgeons would consider GTR to always be the goal, except in situations where resection of a poorly demarcated or infiltrative tumor carries a high risk of neurological injury, cases where there is a heavy burden of metastatic disease, or instances where there are low volumes of residual tumor belonging to highly favorable or unfavorable subgroups. On the other hand, those who advocate a less aggressive approach do so because they feel it lessens the risk of neurological morbidity and allows for less technically demanding surgery while not impacting overall survival and PFS. In the absence of clear evidence, the balance between maximizing extent of resection and minimizing neurological injury is likely influenced by the experience of the individual surgeon and the number of medulloblastomas treated at a particular institution [12]. Therefore, the practical answer to the surgical treatment of this disease may lie within each institution’s experience and comfort level.

## Conclusion

This article reviews the historical and current trends in surgical resection strategy for pediatric medulloblastoma in both North America and other parts of the world. While different surgical strategies may be employed to relatively good effect in separate regions, there is no definitive answer as to best practices. Variation in surgical attitudes is wide, often dependent on surgical volume and experience, as well as resources. This variation in surgical practice coupled with the relative rarity of medulloblastoma and the ever-increasing number of subtypes and treatment regimens makes it very unlikely that a clear understanding of the role of extent of resection will ever be fully achieved. However, as radiographic, diagnostic, and intraoperative technologies continue to improve, surgeons will receive additional valuable information to better counsel families and guide multimodal treatment.

## Data Availability

Not applicable.

## References

[1] Albright AL, Wisoff JH, Zeltzer PM, Boyett JM, Rorke LB, Stanley P (1996) Effects of medulloblastoma resections on outcome in children: a report from the Children’s Cancer Group. Neurosurgery 38:265–271. 10.1097/00006123-199602000-00007 8869053 10.1097/00006123-199602000-00007

[2] Bailey S, Jacobs S, Kourti M, Massimino M, Andre N, Doz F, Dufour C, Vennarini S, Padovani L, Aquilina K, Thomale U, Joshi A, Pietsch T, Avula S, Morana G, Rutkowski S, Pizer B, Clifford SC (2025) Medulloblastoma therapy: consensus treatment recommendations from SIOP-Europe and the European Reference Network. EJC Paediatr Oncol 5:1–18. 10.1016/j.ejcped.2024.100205

[3] Bonifacio-Mundaca J, Casavilca-Zambrano S, Desterke C, Casafont I, Mata-Garrido J (2025) Deciphering medulloblastoma: epigenetic and metabolic changes driving tumorigenesis and treatment outcomes. Biomedicines. 10.3390/biomedicines13081898 40868156 10.3390/biomedicines13081898 PMC12383691

[4] Brandl B, Steiger M, Kubelt C, Rohrandt C, Zhu Z, Evers M, Wang G, Schuldt B, Afflerbach AK, Wong D, Lum A, Halldorsson S, Djirackor L, Leske H, Magadeeva S, Smicius R, Quedenau C, Schmidt NO, Schuller U, Vik-Mo EO, Proescholdt M, Riemenschneider MJ, Zadeh G, Ammerpohl O, Yip S, Synowitz M, van Bommel A, Kretzmer H, Muller FJ (2025) Rapid brain tumor classification from sparse epigenomic data. Nat Med 31:840–848. 10.1038/s41591-024-03435-3 40021833 10.1038/s41591-024-03435-3 PMC11922770

[5] Dubuc AM, Remke M, Korshunov A, Northcott PA, Zhan SH, Mendez-Lago M, Kool M, Jones DT, Unterberger A, Morrissy AS, Shih D, Peacock J, Ramaswamy V, Rolider A, Wang X, Witt H, Hielscher T, Hawkins C, Vibhakar R, Croul S, Rutka JT, Weiss WA, Jones SJ, Eberhart CG, Marra MA, Pfister SM, Taylor MD (2013) Aberrant patterns of H3K4 and H3K27 histone lysine methylation occur across subgroups in medulloblastoma. Acta Neuropathol 125:373–384. 10.1007/s00401-012-1070-9 23184418 10.1007/s00401-012-1070-9 PMC3580007

[6] Dvir R, Elhasid R, Roth J, Constantini S, Peled Y, Ospovat I, Shiran SI (2026) Pediatric metastatic medulloblastoma: upfront biopsy followed by oncological treatment without excision of the primary tumor. J Neurosurg Pediatr. 10.3171/2025.12.PEDS25467 42102399 10.3171/2025.12.PEDS25467

[7] Enayet AE, Nabil M, Rady MR, Yousef Y, Badawy E, El Beltagy MA (2021) Surgical outcome of children with medulloblastoma: a retrospective study of a 405-patient series from Children’s Cancer Hospital Egypt (CCHE-57357). Childs Nerv Syst 37:1931–1940. 10.1007/s00381-021-05082-2 33604717 10.1007/s00381-021-05082-2

[8] Filser M, Torrejon J, Merchadou K, Dufour C, Girard E, Bourneix C, Lemaitre E, Gharsalli T, Brillet R, Wong J, Gentien D, Rapinat A, Servant N, Vasiljevic A, Bertozzi AI, Raimbault S, Tauziede Espariat A, Lhermitte B, Faure-Conter C, Icher C, Berger C, Maurage CA, Bodet D, Meyronet D, Uro-Coste E, De Carli E, Forest F, Palenzuela G, Chotard G, Gauchotte G, Sudour H, Mansuy L, Deparis M, Tallegas M, Faisant M, Entz-Werle N, Varlet P, Leblond P, Michalak-Provost S, Proust Houdemont S, Rigau V, Doz F, Delattre O, Bourdeaut F, Ayrault O, Masliah-Planchon J (2025) Nanopore sequencing as a cutting-edge technology for medulloblastoma classification. Neuro Oncol 27:1313–1324. 10.1093/neuonc/noae279 39731757 10.1093/neuonc/noae279 PMC12187364

[9] Gajjar A, Robinson GW, Smith KS, Lin T, Merchant TE, Chintagumpala M, Mahajan A, Su J, Bouffet E, Bartels U, Schechter T, Hassall T, Robertson T, Nicholls W, Gururangan S, Schroeder K, Sullivan M, Wheeler G, Hansford JR, Kellie SJ, McCowage G, Cohn R, Fisher MJ, Krasin MJ, Stewart CF, Broniscer A, Buchhalter I, Tatevossian RG, Orr BA, Neale G, Klimo P Jr, Boop F, Srinivasan A, Pfister SM, Gilbertson RJ, Onar-Thomas A, Ellison DW, Northcott PA (2021) Outcomes by clinical and molecular features in children with medulloblastoma treated with risk-adapted therapy: results of an international phase III trial (SJMB03). J Clin Oncol 39:822–835. 10.1200/JCO.20.01372 33405951 10.1200/JCO.20.01372 PMC10166353

[10] Hill RM, Richardson S, Schwalbe EC, Hicks D, Lindsey JC, Crosier S, Rafiee G, Grabovska Y, Wharton SB, Jacques TS, Michalski A, Joshi A, Pizer B, Williamson D, Bailey S, Clifford SC (2020) Time, pattern, and outcome of medulloblastoma relapse and their association with tumour biology at diagnosis and therapy: a multicentre cohort study. Lancet Child Adolesc Health 4:865–874. 10.1016/S2352-4642(20)30246-7 33222802 10.1016/S2352-4642(20)30246-7 PMC7671998

[11] Keeling C, Davies S, Goddard J, Ramaswamy V, Schwalbe EC, Bailey S, Hicks D, Clifford SC (2024) The clinical significance of sub-total surgical resection in childhood medulloblastoma: a multi-cohort analysis of 1100 patients. EClinicalMedicine 69:102469. 10.1016/j.eclinm.2024.102469 38374970 10.1016/j.eclinm.2024.102469 PMC10875250

[12] Khan RB, Patay Z, Klimo P, Huang J, Kumar R, Boop FA, Raches D, Conklin HM, Sharma R, Simmons A, Sadighi ZS, Onar-Thomas A, Gajjar A, Robinson GW (2021) Clinical features, neurologic recovery, and risk factors of postoperative posterior fossa syndrome and delayed recovery: a prospective study. Neuro Oncol 23:1586–1596. 10.1093/neuonc/noab030 33823018 10.1093/neuonc/noab030 PMC8408840

[13] Lannering B, Rutkowski S, Doz F, Pizer B, Gustafsson G, Navajas A, Massimino M, Reddingius R, Benesch M, Carrie C, Taylor R, Gandola L, Bjork-Eriksson T, Giralt J, Oldenburger F, Pietsch T, Figarella-Branger D, Robson K, Forni M, Clifford SC, Warmuth-Metz M, von Hoff K, Faldum A, Mosseri V, Kortmann R (2012) Hyperfractionated versus conventional radiotherapy followed by chemotherapy in standard-risk medulloblastoma: results from the randomized multicenter HIT-SIOP PNET 4 trial. J Clin Oncol 30:3187–3193. 10.1200/JCO.2011.39.8719 22851561 10.1200/JCO.2011.39.8719

[14] Louis DN, Perry A, Wesseling P, Brat DJ, Cree IA, Figarella-Branger D, Hawkins C, Ng HK, Pfister SM, Reifenberger G, Soffietti R, von Deimling A, Ellison DW (2021) The 2021 WHO classification of tumors of the central nervous system: a summary. Neuro Oncol 23:1231–1251. 10.1093/neuonc/noab106 34185076 10.1093/neuonc/noab106 PMC8328013

[15] Michalski JM, Janss AJ, Vezina LG, Smith KS, Billups CA, Burger PC, Embry LM, Cullen PL, Hardy KK, Pomeroy SL, Bass JK, Perkins SM, Merchant TE, Colte PD, Fitzgerald TJ, Booth TN, Cherlow JM, Muraszko KM, Hadley J, Kumar R, Han Y, Tarbell NJ, Fouladi M, Pollack IF, Packer RJ, Li Y, Gajjar A, Northcott PA (2021) Children’s Oncology Group Phase III trial of reduced-dose and reduced-volume radiotherapy with chemotherapy for newly diagnosed average-risk medulloblastoma. J Clin Oncol 39:2685–2697. 10.1200/JCO.20.02730 34110925 10.1200/JCO.20.02730 PMC8376317

[16] Mushtaq N, Ul Ain R, Hamid SA, Bouffet E (2023) Evolution of systemic therapy in medulloblastoma including irradiation-sparing approaches. Diagnostics (Basel). 10.3390/diagnostics13243680 38132264 10.3390/diagnostics13243680 PMC10743079

[17] Mynarek M, Milde T, Padovani L, Janssens GO, Kwiecien R, Mosseri V, Clifford SC, Doz F, Rutkowski S (2021) SIOP PNET5 MB trial: history and concept of a molecularly stratified clinical trial of risk-adapted therapies for standard-risk medulloblastoma. Cancers (Basel). 10.3390/cancers13236077 34885186 10.3390/cancers13236077 PMC8657236

[18] Ostrom QT, Price M, Ryan K, Edelson J, Neff C, Cioffi G, Waite KA, Kruchko C, Barnholtz-Sloan JS (2022) CBTRUS statistical report: pediatric brain tumor foundation childhood and adolescent primary brain and other central nervous system tumors diagnosed in the United States in 2014-2018. Neuro Oncol 24:iii1–iii38. 10.1093/neuonc/noac161 36066969 10.1093/neuonc/noac161 PMC9447434

[19] Patel A, Gobel K, Ille S, Hinz F, Schoebe N, Bogumil H, Meyer J, Brehm M, Kardo H, Schrimpf D, Lomakin A, Ritter M, Goller P, Kerbs P, Pfeifer L, Hamelmann S, Blume C, Ippen FM, Berghaus N, Euskirchen P, Schweizer L, Hultschig C, Van Roy N, Van Dorpe J, Van der Meulen J, Loontiens S, Dedeurwaerdere F, Leske H, Halldorsson S, Fox G, Deacon S, Cahyani I, Holmes N, Wibowo S, Munro R, Martin D, Sharif A, Housley M, Goldspring R, Brandner S, Roy S, Hench J, Frank S, Unterberg A, Goidts V, Jager N, Paine S, Smith S, Herold-Mende C, Wick W, Pfister SM, Vik-Mo EO, von Deimling A, Krieg S, Jones DT, Loose M, Schlesner M, Sill M, Sahm F (2025) Prospective, multicenter validation of a platform for rapid molecular profiling of central nervous system tumors. Nat Med 31:1567–1577. 10.1038/s41591-025-03562-5 40133526 10.1038/s41591-025-03562-5 PMC12092301

[20] Patel P, Wallace D, Boop FA, Vaughn B, Robinson GW, Gajjar A, Klimo P (2019) Reoperation for medulloblastoma prior to adjuvant therapy. Neurosurgery 84:1050–1058. 10.1093/neuros/nyy095 29660028 10.1093/neuros/nyy095

[21] Rechberger JS, Power EA, DeCuypere M, Daniels DJ (2024) Evolution of neurosurgical advances and nuances in medulloblastoma therapy. Childs Nerv Syst 40:1031–1044. 10.1007/s00381-023-06239-x 38112693 10.1007/s00381-023-06239-x

[22] Robinson GW, Rudneva VA, Buchhalter I, Billups CA, Waszak SM, Smith KS, Bowers DC, Bendel A, Fisher PG, Partap S, Crawford JR, Hassall T, Indelicato DJ, Boop F, Klimo P, Sabin ND, Patay Z, Merchant TE, Stewart CF, Orr BA, Korbel JO, Jones DTW, Sharma T, Lichter P, Kool M, Korshunov A, Pfister SM, Gilbertson RJ, Sanders RP, Onar-Thomas A, Ellison DW, Gajjar A, Northcott PA (2018) Risk-adapted therapy for young children with medulloblastoma (SJYC07): therapeutic and molecular outcomes from a multicentre, phase 2 trial. Lancet Oncol 19:768–784. 10.1016/S1470-2045(18)30204-3 29778738 10.1016/S1470-2045(18)30204-3 PMC6078206

[23] Schwalbe EC, Lindsey JC, Danilenko M, Hill RM, Crosier S, Ryan SL, Williamson D, Castle J, Hicks D, Kool M, Milde T, Korshunov A, Pfister SM, Bailey S, Clifford SC (2025) Molecular and clinical heterogeneity within MYC-family amplified medulloblastoma is associated with survival outcomes: a multicenter cohort study. Neuro Oncol 27:222–236. 10.1093/neuonc/noae178 39377358 10.1093/neuonc/noae178 PMC11726341

[24] Sharma T, Schwalbe EC, Williamson D, Sill M, Hovestadt V, Mynarek M, Rutkowski S, Robinson GW, Gajjar A, Cavalli F, Ramaswamy V, Taylor MD, Lindsey JC, Hill RM, Jager N, Korshunov A, Hicks D, Bailey S, Kool M, Chavez L, Northcott PA, Pfister SM, Clifford SC (2019) Second-generation molecular subgrouping of medulloblastoma: an international meta-analysis of Group 3 and Group 4 subtypes. Acta Neuropathol 138:309–326. 10.1007/s00401-019-02020-0 31076851 10.1007/s00401-019-02020-0 PMC6660496

[25] Smith AKS, Dhanda SK, Billups CA, Sioson E, Lu C, Peraza AZ, Gangwani K, Li Y, Li Q, Lin T, Michalski JM, Packer RJ, Olson JM, Leary SES, Fouladi M, Gajjar A, Zhou X, Onar-Thomas A, Northcott PA, Robinson GW (2025) An integrated analysis of three medulloblastoma clinical trials refines risk-stratification approaches for reducing toxicity and improving survival. Neuro Oncol. 10.1093/neuonc/noaf250 41159377 10.1093/neuonc/noaf250 PMC12962632

[26] Taylor MD, Northcott PA, Korshunov A, Remke M, Cho YJ, Clifford SC, Eberhart CG, Parsons DW, Rutkowski S, Gajjar A, Ellison DL, Lichter P, Gilbertson RJ, Pomeroy SL, Kool M, Pfister SM (2012) Molecular subgroups of medulloblastoma: the current consensus. Acta Neuropathol 123:465–472. 10.1007/s00401-011-0922-z 22134537 10.1007/s00401-011-0922-z PMC3306779

[27] Thompson EM, Bramall A, Herndon JE 2nd, Taylor MD, Ramaswamy V (2018) The clinical importance of medulloblastoma extent of resection: a systematic review. J Neurooncol 139:523–539. 10.1007/s11060-018-2906-5 29796724 10.1007/s11060-018-2906-5

[28] Thompson EM, Hielscher T, Bouffet E, Remke M, Luu B, Gururangan S, McLendon RE, Bigner DD, Lipp ES, Perreault S, Cho YJ, Grant G, Kim SK, Lee JY, Rao AAN, Giannini C, Li KKW, Ng HK, Yao Y, Kumabe T, Tominaga T, Grajkowska WA, Perek-Polnik P, Low DCY, Seow WT, Chang KTE, Mora J, Pollack IF, Hamilton RL, Leary S, Moore AS, Ingram WJ, Hallahan AR, Jouvet A, Fevre-Montange M, Vasiljevic A, Faure-Conter C, Shofuda T, Kagawa N, Hashimoto N, Jabado N, Weil AG, Gayden T, Wataya T, Shalaby T, Grotzer M, Zitterbart K, Sterba J, Kren L, Hortobagyi T, Klekner A, Laszlo B, Pocza T, Hauser P, Schuller U, Jung S, Jang WY, French PJ, Kros JM, van Veelen MC, Massimi L, Leonard JR, Rubin JB, Vibhakar R, Chambless LB, Cooper MK, Thompson RC, Faria CC, Carvalho A, Nunes S, Pimentel J, Fan X, Muraszko KM, Lopez-Aguilar E, Lyden D, Garzia L, Shih DJH, Kijima N, Schneider C, Adamski J, Northcott PA, Kool M, Jones DTW, Chan JA, Nikolic A, Garre ML, Van Meir EG, Osuka S, Olson JJ, Jahangiri A, Castro BA, Gupta N, Weiss WA, Moxon-Emre I, Mabbott DJ, Lassaletta A, Hawkins CE, Tabori U, Drake J, Kulkarni A, Dirks P, Rutka JT, Korshunov A, Pfister SM, Packer RJ, Ramaswamy V, Taylor MD (2016) Prognostic value of medulloblastoma extent of resection after accounting for molecular subgroup: a retrospective integrated clinical and molecular analysis. Lancet Oncol 17:484–495. 10.1016/S1470-2045(15)00581-1 26976201 10.1016/S1470-2045(15)00581-1 PMC4907853

[29] Toescu SM, Pizer B, Gump W, Aquilina K, Avula S, Mallucci C, Parks C, Carai A, Robinson G, Aldave G, Baird L, Niazi T, Keating R, Klimo P Jr. (2025) Toward reducing the risk of cerebellar mutism syndrome: consensus statement from the Posterior Fossa Society. J Neurosurg Pediatr 36:789–797. 10.3171/2025.5.PEDS2593 41072042 10.3171/2025.5.PEDS2593

[30] Wells EM, Khademian ZP, Walsh KS, Vezina G, Sposto R, Keating RF, Packer RJ (2010) Postoperative cerebellar mutism syndrome following treatment of medulloblastoma: neuroradiographic features and origin. J Neurosurg Pediatr 5:329–334. 10.3171/2009.11.PEDS09131 20367335 10.3171/2009.11.PEDS09131

[31] Zeltzer PM, Boyett JM, Finlay JL, Albright AL, Rorke LB, Milstein JM, Allen JC, Stevens KR, Stanley P, Li H, Wisoff JH, Geyer JR, McGuire-Cullen P, Stehbens JA, Shurin SB, Packer RJ (1999) Metastasis stage, adjuvant treatment, and residual tumor are prognostic factors for medulloblastoma in children: conclusions from the Children’s Cancer Group 921 randomized phase III study. J Clin Oncol 17:832–845. 10.1200/JCO.1999.17.3.832 10071274 10.1200/JCO.1999.17.3.832

